# It’s Not Just Trump: Americans of Both Parties Support Liberal Democratic Norm Violations More Under Their Own President

**DOI:** 10.1093/poq/nfae042

**Published:** 2024-10-23

**Authors:** Levente Littvay, Jennifer L McCoy, Gabor Simonovits

**Affiliations:** Research Professor, HUN-REN Centre for Social Sciences, Budapest, Hungary; and Senior Research Fellow, Democracy Institute, Central European University, Budapest, Hungary; Regent’s Professor, Department of Political Science, Georgia State University, Atlanta, GA, US; Associate Professor, Department of Political Science, Central European University, Vienna, Austria; and Senior Research Fellow, HUN-REN Centre for Social Sciences, Budapest, Hungary

## Abstract

There is a growing worry about the health of American democracy, and political scientists and pundits alike are looking for possible explanations. Surveys conducted during the Trump presidency showed considerable citizen support for liberal democratic norm erosions, especially among Republicans. However, recent experimental research also shows that voters of both parties are more tolerant of norm erosion committed by politicians of the party they prefer. In this note, we aim to reconcile these contradictory findings by analyzing surveys spanning from 2006 to 2021 on the public’s tolerance of executive concentration of power. We also collect original data under both the Trump and Biden administrations gauging support for a broad array of liberal democratic norm erosions. Support for such erosions, in fact, has been relatively similar across Democrats and Republicans once we account for the party of the president. Support for executive aggrandizement has been prevalent among supporters of the president’s party at least since the second term of the Bush administration. Increased checks and balances on the executive, through divided government, amplifies this effect further. Taken together, these findings suggest that universal support for the liberal democratic status quo has been weaker among those who support the president’s party, well before and since the Trump presidency.

## Introduction

Americans say they want democracy,^1^ yet they continue to support political behavior that is eroding existing democratic norms—the unwritten rules that guide behavior in a particular democratic society. This study provides evidence that partisans’ support for liberal democratic norms has been contingent on whether one’s own party controls the presidency. This pattern is generalizable across multiple indicators of norm erosion, and across all presidential administrations between 2006 and 2021. Evidence on the prevalence of, and partisan differences in, norm erosion has been elusive because political scientists have only recently started to measure support for *specific* instances of democratic norm erosion, and because much of what we know about this is from studies conducted during the Trump administration. The Trump era spurred a growing interest among scholars to explore the degree to which the public can be counted on to stand up as a guardian of democratic norms and institutions when political elite behavior threatens them (Foa and Mounk 2017; Svolik 2019; Claassen 2020; Graham and Svolik 2020; Gidengil, Stolle, and Bergeron-Boutin 2021; Carey et al. 2022; Simonovits, McCoy, and Littvay 2022; Tai, Hu, and Solt 2024; Wuttke, Gavras, and Schoen 2022; Krishnarajan 2023).

Trump-era studies found that support for norm violations was more prevalent among Republicans than Democrats (Drutman, Diamond, and Goldman 2018a, 2018b; Drutman, Goldman, and Diamond 2020; Gidengil, Stolle, and Bergeron-Boutin 2021). These partisan differences could reflect cue taking from Trump’s unbridled espousal of unrestrained executive authority (Barber and Pope 2019; Bartels 2020; Gidengil, Stolle, and Bergeron-Boutin 2021; Kingzette et al. 2021); they could also come from individual differences in ideology (Malka et al. 2022; Svolik et al. 2023) or personality traits (Hetherington and Weiler 2009). But a simpler explanation is that greater support for backsliding among Republicans during the Trump era was found simply because a Republican was in power.

Relying on hypothetical candidate choice experiments, Graham and Svolik (2020) already found that voters of both parties are more likely to condone the violations of democratic norms when these are committed by their preferred candidates. In fact, Simonovits, McCoy, and Littvay (2022) found in survey experiments that democratic violations are explicitly supported more when one’s preferred party is, hypothetically, in power, something they called *democratic hypocrisy.*^2^ The problem with the existing studies, however, is that they are either based on hypotheticals limiting their external validity and making it difficult to assess real support for real policies endorsed by real candidates, or they are based on cross-sectional observational studies in which the context, such as who is the president in office at the time of the study, is fixed. To overcome this issue, we turn to longitudinal data across multiple administrations and multiple indicators of norm violations in an original data collection during the Trump and Biden administrations. Our design also allows us to explore the impact of divided governments, especially in the presence of a survey item directly tapping separation of powers.

## Data and Methods

We compare support for specific norm-eroding policies across Democrats and Republicans at several different points in time based on two datasets. First, we rely on survey data collected by the Americas Barometer project, a comparative survey designed to understand the fragile democracies of Latin America, but also collected in the United States (AB). Second, we conducted original surveys in August 2020 (n = 1,559) and December 2021 (n = 1,058) to obtain a more fine-tuned picture of democracy eroding policy support. Below, we summarize both datasets.

The AB surveys ask about the elimination of congressional checks and balances on the president, egregious violations of existing liberal democratic norms in the United States. This allows us to assess norm erosion in a longer time series under four different US presidents, two Republicans and two Democrats.^3^ In its first wave of 2006, the AB data includes 609 respondents recruited through random-digit dialing. In subsequent waves—fielded in April 2008, the second half of March 2010, April 2012, June–July 2014, May 2017, July 2019, and June–July in 2021—sample sizes amounted to 1,500 respondents and were fielded by YouGov/Polimetrix. In our main analysis, we rely on the following item: “Do you think that sometimes there could be enough justification for the President to shut down Congress or do you think there can never be sufficient justification to do so?” In Supplementary Material figures SF3 and SF4, we demonstrate similar patterns based on two additional questions appearing less consistently in AB waves.^4^

To complement the AB data with a broader set of measures describing support for liberal democratic norm violations in the US context, we fielded our own surveys before and after a presidential (and congressional) power shift in 2020. Data collection took place in August 2020 (n = 1,559) and December 2021 (n = 1,058) through Lucid, the largest US marketplace for online panels. While Lucid ensures that samples resemble the US population age groups, gender, race, and region, and Coppock and McClellan (2019) showed that findings derived on Lucid samples approximate well findings with higher-quality surveys, our original data is by no means a probability sample. For this reason, we use these results only to complement our analyses of the AB data and interpret them with caution. To enhance data quality, we screened out inattentive respondents using an attention check early in the questionnaire (Berinsky, Margolis, and Sances 2014).^5^

To get a more fine-tuned picture of democracy eroding policy support, we designed sixteen questions to study norm erosion of the liberal democratic status quo in the US context balanced across four components of liberal democracy: majority rule (mr), executive restraint (er), civil liberties (cl), and rule of law (rl). The questions are designed to assess willingness to change the prevailing democratic norms for the last half century in the United States (even where some of these have changed within the last decade in some states or cases), rather than to be absolute measures of levels of democracy. Some behaviors can be considered democratizing in some national contexts and de-democratizing in another, as they change the balance between liberal and majoritarian aspects of democracy. Our items range from policy choices that are constitutional but shift prevailing norms of behavior, to more egregious violations of democratic principles and laws.

For example, expanding the Supreme Court is considered in many democracies to be a means to advance an incumbent’s preferred policies. Yet, in the United States, the expansion of the court could even be seen as a liberal democratic corrective of an unfairly achieved partisan advantage on the court: the Republican Party denied Obama a nominee (Garland) in 2016, arguing that voters could soon decide who should pick the replacement in that year’s election, but then approved Trump’s nominee (Barrett) three weeks before the 2020 election. On the other side of the spectrum, the items include clearer violations of constitutional rights, such as banning protests, or violations of the rule of law, such as ignoring court rulings. They were designed to reflect realistic examples from gradual to more extreme norm violations to test the respondents’ tolerance across a range of policies. The wording and explanation of these questions can be found in the Supplementary Material.

## Results

**Figure 1.**
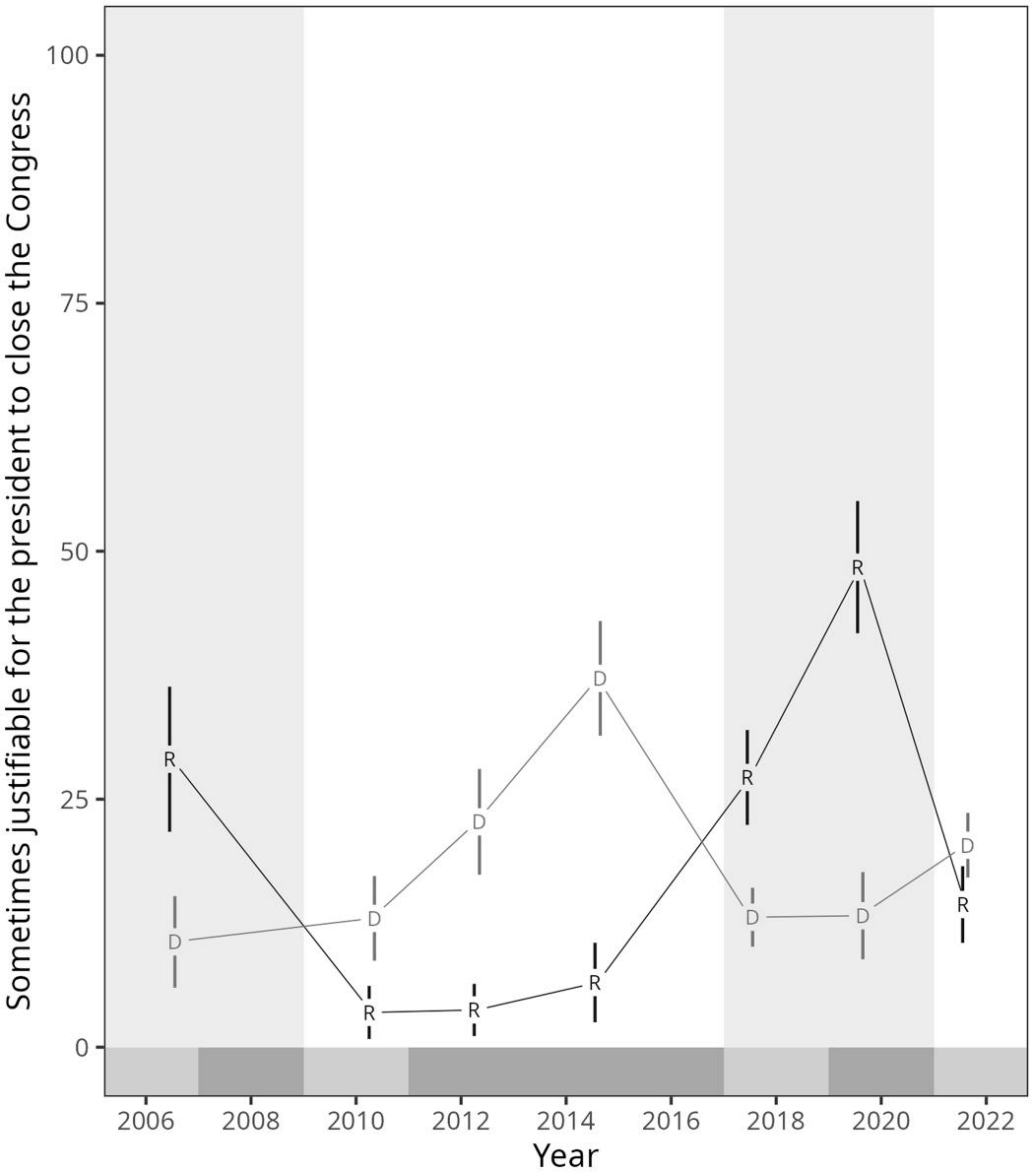
Americas Barometer data for the United States between 2006 and 2021. Republican administrations with gray background, Democratic ones with white. Divided governments are denoted under the origin on the X-axis, where darker marks show divided governments and lighter marks unified governments. Data points were replaced with D for Democrats and R for Republicans. Error bars denote +/− 2 standard errors approximating 95 percent confidence intervals. Data are weighted using sampling weights provided by AB. Numerical information is provided in Supplementary Material table ST1.

We first describe our findings based on the Americas Barometer. Figure 1 plots the percentage of Republicans and Democrats that agreed with the statement that “it is sometimes justifiable for the president to shut down the Congress.”^6^ The results clearly show that identifiers of the party of the president in power are consistently more likely to approve of this action. While the strongest approval for such an action is observed among Republicans under the second half of Donald Trump’s term, perusing the longer time span reveals no systematic differences *across* Democrats and Republicans either when in or out of power. Other measures of democratic norm violations available for both Democratic and Republican administrations, although for fewer waves, lead to qualitatively similar conclusions and are presented in Supplementary Material figures SF3 and SF4.

Regression analysis presented in table 1 shows that those whose preferred president is in power approve of them dissolving Congress 15.8 percent more (*p *= 0.011), on average, than those whose preferred party is out of power. Exploring potential factors driving the year-to-year variation in this effect seen in figure 1, we also tested and found that this difference in support between Republicans and Democrats more than doubled under a divided government. The gap in support between the parties under a divided government is an additional 16.5 percent greater (*p *=* *0.033).

**Table 1.** Regression analysis of support for dissolving Congress and divided government.

	Dependent variable:	
	Justifiable to dissolve Congress	
	(1)	(2)
Respondent and president have same party	15.779	10.010
	*p* = 0.011	*p* = 0.020
Divided government		−3.046
		*p* = 0.423
Same party x divided government		16.541
		*p* = 0.033
Intercept	10.781	11.777
	*p* = 0.002	*p* = 0.006
Observations	3,937	3,937
Log likelihood	−20,203.38	−20,173.32
Akaike Inf. Crit.	40,410.76	40,354.65

*Note*: Standard errors are clustered at the level of survey waves.

We next present corresponding findings based on our original data collection. Figure 2 presents the change in the support for the sixteen liberal democratic norm erosion items by party.^7^ Our results demonstrate the pattern consistent with democratic hypocrisy, where Democrats tended to become more supportive of norm erosion after Biden’s election and support for most of these measures declined among Republicans from 2020 to 2021. Averaging support for norm erosion across all sixteen items reveals that the partisan differences accounted for about one-quarter (3.0 percent) of the change in the attitudes across administrations for both Republicans (−13.6 percent) and Democrats (+10.6 percent) highlighting the effect’s magnitude. Republican support for the sixteen erosion items was, on average, only slightly higher than Democrats’ (34.1 percent vs 31.1 percent).

**Figure 2.**
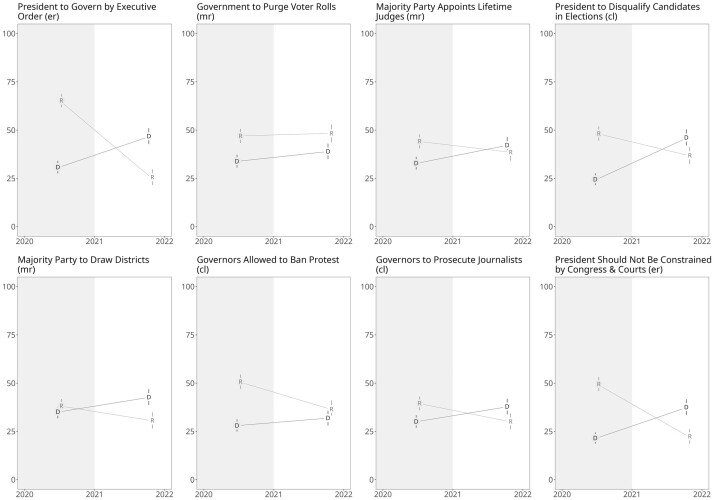
Change in the support for liberal democratic norm erosion between 2020 under Trump and 2021 under Biden. Trump (R) administrations with gray background, Biden (D) administration with white. Data points were replaced with D for Democrats and R for Republicans. Error bars denote +/− 2 standard errors approximating 95 percent confidence intervals. Data are weighted using sampling weights provided by AB. Figure is ordered (descending) by average level across parties and time points. Numerical information is provided in Supplementary Material table ST2.

**Figure 2.**
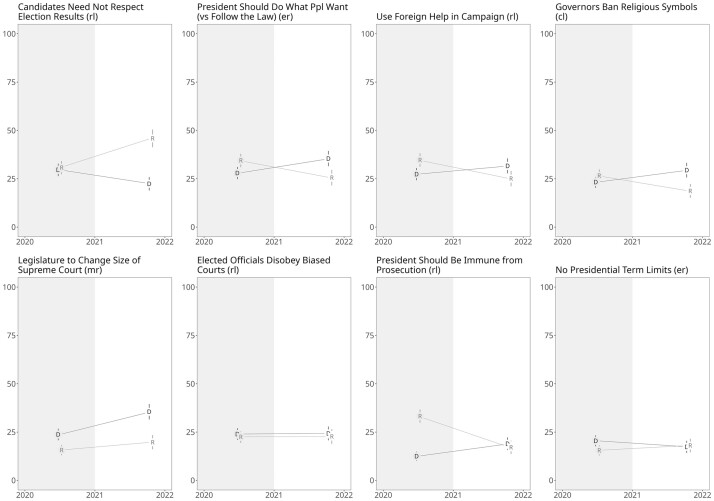
(Continued).

The most striking exception to this overall pattern was that Republican respondents in 2021 grew even more willing to accept candidates’ rejection of election results. Regardless of who is controlling the presidency, Republicans agreed more with this position. The Democrats, on the other hand, were more approving of changing the size of the Supreme Court in both years. We note that a majority of respondents in both surveys rejected the norm erosion position on all items except for governing by executive order, so that we should be careful not to overstate the implications of these findings and conclude that Americans are willing to give up democracy entirely.

## Discussion

Within a worldwide democratic recession, the United States stands out among Western liberal democracies for its recent backsliding. Numerous expert rankings document the erosion of liberal democracy in the United States already since 2010, and in an accelerated manner since the election of Trump in 2016 (Coppedge et al. 2020; Repucci 2022; Economist Intelligence Unit 2022). Several analyses have pointed to an asymmetry between Democratic and Republican party leaders. Experts find that the Republican Party by 2019 had become more similar to autocratizing parties in Turkey, India, and Hungary than to mainstream conservative parties in Europe, in that it opposed checks and balances on executive power and undermined liberal democratic principles (Norris 2020).^8^ Leaders espoused antipluralist rhetoric: defined as demonizing political opponents, disrespecting fundamental minority rights, and encouraging political violence (Lührmann et al. 2020).^9^ A study of democratic indicators in all 50 US states from 2000 to 2018 found that Republican control of state government dramatically reduced states’ democratic performance during this period (Grumbach 2022).

Unfortunately, our results from both studies show that the public, especially those with their preferred parties in power, cannot necessarily be counted on to definitively counterbalance these trends. The people also will not objectively protect liberal democratic rights and liberties that may be threatened by political elites. While leading intellectuals made sweeping claims about the greater proclivity of Republican voters to support democracy-eroding policies and showcased this as a novel tendency attributable to the emergence of Donald Trump, our empirical findings suggest otherwise. Looking at it across administrations, differences between Democrats and Republicans are found to be modest, as the supporters of both parties are more willing to look the other way when their own party leaders act uninhibited by existing norms. Nonnegligible numbers of Democratic and Republican voters, at least since 2006, have prioritized partisan preferences over democratic principles even if, on the elite level, Democrats are less norm eroding. This is especially the case for majoritarian power abuses and to a lesser extent violations of civil liberties. Results are mixed for policies that would lift institutional restraints on the president, while rule-of-law violations are generally less supported.

Most threatening to democracy are those supporting the idea of executive aggrandizement—a key indicator of democratic erosion—and those supporting losing candidates’ rejection of election results, undermining a foundational expectation of democratic commitment. Not surprisingly, in an era of perceived partisan gridlock, the highest level of support in our surveys was to give the president more leeway to act without constraints from Congress: the use of executive orders. But the support across the parties for their own president to potentially close Congress or operate without congressional or judicial constraints represents a clear challenge to the balance of powers enshrined in the US Constitution and is reminiscent of executive coups in Latin America (Orbay 2022). The willingness to support rejecting election results across both parties—reaching a near majority among Republican respondents, but also 20–30 percent among Democrats—indicates the degree to which the contentious debate over election integrity and fairness roils the American polity. The 2022 midterm election results, in which most election-denier candidates for Senate and statewide offices were defeated, is a sign that voters may be rejecting the most extreme positions. Yet the closeness of many of these races, and the large numbers of election deniers elected to state legislatures and the House of Representatives, indicates that these positions still pose a threat.

The long time span of the observational data on the support for the president to, at times, close Congress also offers a hint regarding the drivers of support for executive aggrandizement. Partisan differences have been consistently greater in years when the government was divided (2012, 2014, 2019) and much smaller in periods when the same party held the presidency and the two houses of Congress (with the possible exception of 2006). For instance, during the Obama presidency, support for the statement rose rapidly among Democrats after Republicans took over the House in 2011 and the partisan gap increased even further by the end of the Obama years when Republicans, now in the majority in the Senate beginning in 2015, refused to even consider Obama’s Supreme Court nominee. Similarly, while Republican support for shutting down Congress increased abruptly once Trump took office even with both chambers controlled by Republicans, it *doubled* by 2019, once the House of Representatives was taken by the Democrats.

Taken together, these patterns suggest that a possible cause of both the levels and variation in the opposition to liberal democratic norms is simply a disenchantment of voters about the inability of the executive to bring about political action. Frustrations of a divided government may lead the supporters of the president to want to dismantle checks on executive action, highlighting a democracy-eroding property of divisions of power. The fact that we observe large partisan differences even in years when the same party held the two chambers and the presidency suggests that these frustrations may also have grown as the filibuster was increasingly used by the minority party in the Senate to block presidential appointments and major legislation over the course of this entire time period. But alternative explanations are also possible, such as that presidential support for democracy erosion accumulates throughout an administration as memories of what life was like under an opposing president fade. For example, figure 1 shows a consistent increase in the gap under both the Obama and Trump administrations. The relatively wide gap in 2006, during Bush’s second term and despite a unified government, offers some evidence of this as well.

The literature on democratic backsliding has also pointed to other explanations. Perhaps most prominently, large comparative macro-level studies point to pernicious political polarization—the division of society into mutually distrustful camps—as a main culprit of popular support for democracy-eroding leaders and eroding citizen support for liberal democratic norms (McCoy and Somer 2018, 2019; Carothers and O’Donahue 2019; Finkel et al. 2020; Haggard and Kaufman 2021). The argument that the declining respect for institutions of power sharing is harmed by perceptions of mutual existential threat is also corroborated by micro-level studies (Finkel et al. 2020; Graham and Svolik 2020; Simonovits, McCoy, and Littvay 2022) and demonstrates that citizens rationalize their perceptions of democratic behavior based on their policy preferences (Krishnarajan 2023).

Studies focusing on *affective* polarization among individuals (like of in-party and dislike of out-party) show mixed results, with some finding a relationship between affective polarization and antidemocratic attitudes (Lelkes and Westwood 2017; Kingzette et al. 2021), while others conclude that interventions aimed to reduce partisan animosity do not necessarily reduce anti-democratic attitudes (Broockman, Kalla, and Westwood 2023; Voelkel et al. 2023).

Unfortunately, even with the data at hand—which encompasses a much longer time period than earlier studies—we are not in a position to speak to this debate. It is possible that in earlier times lower polarization coincided with greater support for liberal democratic norms no matter which party was in power. In order to even attempt causal explanations, future studies should compare democratic hypocrisy in countries with low polarization to determine to what extent it is caused by extreme political polarization, or is a natural feature of democracy.

Another direction for future research stems from the nascent literature examining the relationship between an individual’s preferred type of democracy (majoritarian, liberal) or nondemocracy (autocrats) and their support for norm violations. But so far, this literature has only considered individual-level factors such as policy preferences and demographics (Krishnarajan 2023). Our study, on the other hand, finds that context also matters, and matters quite heavily. Attitudes may shift as the party in power shifts. This could be due to growing perceptions of threat, dissatisfaction with government performance, or some other cause, but ultimately derives from contextual and not individual factors. Further research into the impact of heterogeneous views of democracy, and the stability of democratic attitudes (conceptualized diversely) and studied over time using longitudinal panel data, could help clarify this.

Our study contributes to the growing literature raising the alarm about democratic vulnerability in the United States, and points to the urgent need for further research on potential mitigation strategies to restore democratic resilience, to depolarize, and to increase elite commitment to a healthy competition in the marketplace of political ideas. From the extensive literature in political psychology, social psychology, social identity theory, and inter-group conflict, we may devise new narratives and mechanisms to reassure voters and reduce perceptions of mutual existential threat and distrust. From comparative political science, we should investigate electoral and other institutional reforms that may change the incentives and behavior of voters and parties alike (Drutman 2020). Finally, comparative historical analysis points to the important role of social movements, broad sectoral coalitions, and grassroots community projects in innovating and pressing for broad political reform (Putnam and Garrett 2020; Tarrow 2021, p. 2).

## Supplementary Material

nfae042_Supplementary_Data

## Data Availability

Replication data and documentation are available at https://doi.org/10.7910/DVN/SBBOUQ.

## Footnotes

1 Studies of support for democracy are based on vague survey questions. For instance, studies such as the World Values Survey produced strong general endorsement of democracy in the United States with generally above 80 percent saying democracy was a fairly good or a very good system between 1995 and 2018 (see Supplementary Material figures SF1 and SF2).

2 Comparative research also shows that a similar phenomenon exists in other countries and political contexts (Albertus and Grossman 2021; Aarslew 2022; Goodman 2022; Mazepus and Toshkov 2022; Şaşmaz, Yagci, and Ziblatt 2022; Cohen et al. 2023). In their US study, Graham and Svolik (2020) called these people “conditional democrats,” but we continue to use the term “democratic hypocrisy.”

3 This item was on the questionnaire in 2006 (Bush—R), 2010 (Obama—D), 2012 (Obama—D), 2014 (Obama—D), 2017 (Trump—R), 2019 (Trump R), and 2021 (Biden—D).

4 “Do you think that sometimes there could be enough justification to shut down the Supreme Court or do you think that there can never be sufficient justification to do so?” in 2006, 2010, 2012, and 2019 measured with the same dichotomous item as figure 1, and “It is necessary for the progress of this country that our presidents limit the voice and vote of opposition parties, how much do you agree or disagree with that view?” collected in 2008, 2010, and 2012 and measured on a 7 point scale from Strongly Disagree to Strongly Agree.

5 Unfortunately, because surveys fielded both through Lucid and YouGov/Polimetrix rely on panelists, and because we cannot observe everyone who considered taking the survey, we cannot compute response rates. More information about the sampling design of AB surveys we use is available here: https://www.vanderbilt.edu/lapop/united-states.php

6 To determine partisanship, the following question was used: “Which political party do you favor?,” which was updated to “Which political party do you identify with?” in 2010 and 2012, and to “Generally speaking, do you think of yourself as a Republican, a Democrat, an Independent, or what?” from 2014. Independents, people citing other parties or uncertainty, and refusals were excluded from the analysis.

7 To classify Republicans and Democrats, the classic ANES party ID question was used. We classified leaners as partisans, and pure Independents and third-party supporters were also forced to choose between the two major parties through a dichotomous forced-choice question on which party deals better with “issues most important to” them. In Supplementary Material figure SF5, we show that our results remain the same if we exclude Independents and third-party supporters.

8 The Global Party Integrity 2019 index rated the Republican Party about 8 on a 0–10 scale, with 10 being the most undermining of these principles and 0 the most respectful. The Democratic Party was rated about 2 on each principle.

9 The V-Party database produced by the V-Dem Institute found that the Republican Party increased from about 0.25 in 2000 to about 0.7 in 2018 on the 0–1 Illiberalism scale, while the Democratic Party remained at 0.1 from 2000 to 2018.
