# Supplementary material for: It’s Not Just Trump: Americans of Both Parties Support Liberal Democratic Norm Violations More Under Their Own President
Source: Public Opin Q. 2024 Oct 23;88(3):1044–58. doi: 10.1093/poq/nfae042 (PMC11664217; doi:10.1093/poq/nfae042)
Supplement: nfae042_Supplementary_Data [file nfae042_supplementary_data.pdf]

Supplementary Material: It's not just Trump: Americans of both parties support liberal democratic norm violations more under their own president. (Published in *Public Opinion Quarterly*)*

Levente Littvay[1,2], Jennifer L. McCoy[3,2], and Gabor Simonovits[4,1]

[1]HUN-REN Centre for Social Sciences, Institute for Political Science, Budapest, Hungary
[2]Central European University, Democracy Institute, Budapest, Hungary
[3]Georgia State University, Department of Political Science, Atlanta, Georgia, USA
[4]Central European University, Department of Political Science, Vienna, Austria

Table of Contents

Supplementary Text: Original Survey Question Wordings. 2
Supplementary Figures: Support for Democracy - World Values Survey 1995 – 2018 6
Supplementary Figures and Table: Additional Americas Barometer Results (with fewer waves) 7
Supplementary Tables & Figures: Original Data Collection Figure (2) w/ Independents and Numerical Information 10

List of Figures

Supplementary Figure SF1 Support for Democracy - World Values Survey 1995 – 2018 6
Supplementary Figure SF2 Support for Democracy - World Values Survey 1995 – 2018 by Party 6
Supplementary Figure SF3 Latin America Barometer: Justifiable to Close Supreme Court 7
Supplementary Figure SF4 Latin America Barometer: Can Limit the Voice & Vote of the Opposition 8
Supplementary Figure SF5: Original Data Collection Figure 2 but Including Independents. 11

List of Tables

Supplementary Table ST1: Numerical Information for Americas Barometer Figures 9
Supplementary Table ST2: Numerical Information for Figure 2 in Main Text 10
Supplementary Table ST3: Numerical Information for Figure SF5 12

*For correspondence, please e-mail Gabor Simonovts at simonovitsg@ceu.edu

Supplementary Text: Original Survey Question Wordings.

(Norm eroding responses in bold.)

Legislature to Change Size of Supreme Court (mr) Some people say that the majority in the legislature should be able to change the number of justices on the Supreme Court, even if the opposing party disagrees. Others say that the Supreme Court should stay at the current size of nine justices, regardless of their current ideology.

What do you think?

- o Supreme Court size should stay the same
- o **The Senate should change the number of justices to swing its ideology**

Interpretation: *in most countries and most eras, expanding the size of the Supreme Court is interpreted as court-packing to advantage the incumbent's policies or interests. In the U.S. the constitution allows the Congress to change the size, as it has done multiple times in its history. At the time of the survey, the proposal was interpreted in a polarized fashion, however, with Democrats often arguing it would be democratizing to expand the Court to balance out the partisan imbalance following Republican refusal to hold a hearing and vote on Barack Obama's nominee in the election year of 2016, filling it in the next term, and then approving Donald Trump's nominee three weeks before the 2020 election. Republicans tended to view discussion of court expansion as partisan court-packing. We code the norm-eroding position as "The Senate should change the number of justices to swing its ideology."*

Majority Party Appoints Lifetime Judges (mr) The Constitution does not require it, but the Senate has historically operated on a principle of consulting the minority party to make lifetime judicial appointments like Supreme Court and federal judges. It did this through the filibuster rule requiring 60% approval to hold a vote on an appointment, although the appointment vote itself only requires a 51% approval.

In the last 8 years, though, the Democrats and Republicans have each reduced the filibuster so that currently, the majority party alone is able to appoint lifetime judges, including the Supreme Court. Some people say it is right that the majority party in the Senate should be able to make lifetime judicial appointments with their own 51% majority, without any bipartisan consensus. Others say that the filibuster should be restored in order to encourage the practice of bipartisan consensus for these appointments.

Which statement do you agree with more?

- o **Allow majority party to appoint lifetime judges.**
- o Require bipartisan consensus to appoint judges.

Interpretation: *the norm of behavior for most of the 20th century into the 21st century was to require a filibuster super-majority of 60 votes to consider lifetime appointees, with the intent to ensure some level of bipartisan consensus on such important positions. It was only in the last decade that that norm changed. We coded the norm-eroding response as "Allow majority party to appoint lifetime judges."*

Majority Party to Draw Districts (mr) The constitution allows the states to decide who will draw the voting districts.

Some people say that whichever party is in the majority in each state should be able to draw voting district lines that give them additional seats in the legislature. And others say that a non-partisan independent body should draw voting district lines to eliminate advantages to one party or the other.
In your opinion who should be in charge of redistricting?

- o **The majority party**
- o An independent commission

Interpretation: *while the U.S. states are divided as to whether they use partisan or non-partisan bodies to draw district lines, the question refers to giving partisan advantages through additional seats in the legislature. We coded this as norm-eroding as it refers not just to who should draw lines, but whether the majority party should be intentionally advantaging their own party.*

Government to Purge Voter Rolls (mr) Some people say that the state government should clean up the voter rolls by removing voters if they have not voted in the last two elections. Others say that it is a constitutional right to vote and voter names should not be removed once they have registered.

What do you think should happen with the names of those who do not vote regularly?

- o **They should be removed from the roll**
- o They should stay on the roll

Interpretation: *states have a responsibility to keep voter rolls updated by removing deceased voters or those who have moved; however, the question refers specifically to removing voters because they have not voted in two recent elections, which had not been a common practice but began to be practiced in some states after 2015. Thus we coded as norm-eroding "They should be removed from the roll."*

President to Govern by Executive Order (er) Legally, the president has authority to write executive orders to enforce laws and implement policy. These orders may be overturned by the courts if they are determined to violate existing law, or if the Congress votes against them.

Some people say that the president should be able to change important national policy, like healthcare or immigration, by using the powers of executive order when Congress refuses to cooperate. Others say that only the Congress should be able to make major changes to national policy.

Should the president be able to make major changes to policy without the consent of Congress?

- o **Yes, the president should**
- o No, the president should not

Interpretation: *executive orders have grown in recent years with Congressional gridlock, but have also been challenged in courts and criticized or overturned by the opposing party when they return to a majority. The question refers to the president being able to unilaterally change important national policies, seen as executive aggrandizement and counter to the separation of powers, and thus we coded as norm-eroding "Yes, the president should be able to make major changes to policy without the consent of Congress."*

President Should Do What Ppl Want (vs Follow the Law) (er) Some people say that the president should do what the people want even if it goes against existing laws. Others say that the president should follow the law even if it's not what the people want.

In your opinion, should the president do what the people want even if it goes against the law?

- o **President should do what people want**
- o President should follow the law

Interpretation: *this is more clearly a practice of executive aggrandizement to go against existing laws, and thus we coded as norm-eroding "President should do what people want even if it goes against the law".*

President Should Not Be Constrained by Congress and Courts (er) Some people say that our president should have the necessary power to act in favor of the national interest, even if Congress or the Supreme Court opposes it. Others say that the president should get the agreement first from Congress and the Supreme Court before making major changes.

Which of the following statements you most agree with.

- o **The President should have the power to act alone**
- o President should get agreement of Congress and Supreme Court

Interpretation: *this provision violates the norm of separation of powers in the U.S. No Presidential Term Limits (er) Some people say that term limits are undemocratic because they do not allow the people to keep voting for a popular president. Others say that term limits are needed to make sure no single person gains too much power over the country.*

No Presidential Term Limits (er) Some people say that term limits are undemocratic because they do not allow the people to keep voting for a popular president. Others say that term limits are needed to make sure no single person gains too much power over the country.

On the whole, are you in favor or opposed to presidential term limits?

- o Favor presidential term limits
- o **Oppose presidential term limits**

Interpretation: *an argument could be made that removing term limits is the most democratic position, as strongmen have argued such as Daniel Ortega in Nicaragua or Hugo Chavez in Venezuela. In the United States, a two-term limit was added after Franklin Delano Roosevelt's 4th term and has been the norm in the U.S. for over 70 years, thus we coded as norm-eroding "Oppose presidential term limits".*

Governors Allowed to Ban Protest (cl) Do you think that governors should be allowed to ban protests, or is it more important to defend the right to protest, even by extremists?

- o Governor should NOT be allowed to ban protests
- o **Governor should be allowed to ban protests**

Interpretation: *giving the governor the right to ban protests goes against constitutional protections of free speech and freedom of assembly*

Governors to Prosecute Journalists (cl) Imagine that the governor of your state is pushing the authorities to prosecute a journalist who accused the governor of misconduct. In this scenario, should the governor have the right to prosecute this journalist?

- o **Yes, governor should have the right to prosecute**
- o No, governor should not have the right to prosecute

Interpretation: *prosecuting journalists for accusing a governor of misconduct goes against the norm of protection of free speech in the U.S.*

Governors Ban Religious Symbols (cl) In your opinion, should governors have the right to ban people from wearing things that express religious affiliation in public?

- o **Yes, governor should have that right**
- o No, governor should not have that right

Interpretation: *wearing of religious symbols are protected by the first amendment and banning it goes against freedom of expression.*

Elected Officials Disobey Biased Courts (rl) Some people say that elected officials must obey the courts even when they think that the decisions are politically biased against the president's party, while others argue that elected officials should not be bound by court decisions they regard as biased.

What do you think?

- o Court rulings should always be obeyed
- o **Some court rulings should be disregarded**

Interpretation: *disregarding court rulings on arbitrary bases violates the norms of rule of law and separation of powers as practiced in the U.S.*

Use Foreign Help in Campaign (rl) Some people say that candidates should be able to use any information about their opponents during the campaign, even if it comes from outside the country and is difficult to verify. Others say that the new Congress should pass a law requiring all candidates to report to the FBI any foreign offers of help, such as dirty information on their opponents.

In your view, should foreign information be used in campaigns or reported to the FBI?

- o **It should be used in the campaign**
- o It should be reported to the FBI

Interpretation: *foreign financial contributions to campaigns is illegal in the U.S., while using dirty unverifiable information is a gray area. This became an issue in the 2016 election as Trump accused Clinton of using an unverified dossier of information from Russia against him, while Clinton accused Trump of encouraging the Russian leak of Clinton and Democratic Party emails against her. We coded as going against norms of behavior in the U.S. "It should be used in the campaign".*

Candidates Need Not Respect Election Results (rl) Some people say that political candidates should respect election results even if they believe they lost an election due to unfair practices. Others say that they should refuse to accept results that they believe are due to unfair practices, even if they can't prove it conclusively.

What do you think candidates should do when they think they lost due to unfair practices?

- o Accept the results
- o **Refuse to accept the results**

Interpretation: *it violates norms of behavior and law in the U.S. to refuse to accept election results without proof of unfair practices that changed the results.*

President to Disqualify Candidates in Elections (cl) Do you think the president should have the power to disqualify candidates he believes to be disloyal to the country?

- o **Yes, president should have the right to disqualify candidates**
- o No, president should not have the right to disqualify candidates

Interpretation: *it is unconstitutional for the president to determine who can run for office.*

President Should Be Immune from Prosecution (rl) Some people say that the president is serving the country and therefore should be immune from prosecution for any action he/she takes as president. Others say that no one is above the law and the president should be investigated in an impeachment inquiry, and removed from office if found guilty.

In your view, should the president be impeached and removed for such behavior or should be immune during his or her presidency?

- o Be impeached and removed
- o **Enjoy immunity**

Interpretation: *while immunity from prosecution by the Justice Department while in office is debated among legal scholars, the role of Congress to investigate, and potentially impeach and remove a president, as described in the question, is constitutionally protected in the separation of powers and oversight. Thus we coded "Enjoy immunity" as norm-eroding.*

Supplementary Figures: Support for Democracy - World Values Survey 1995 – 2018

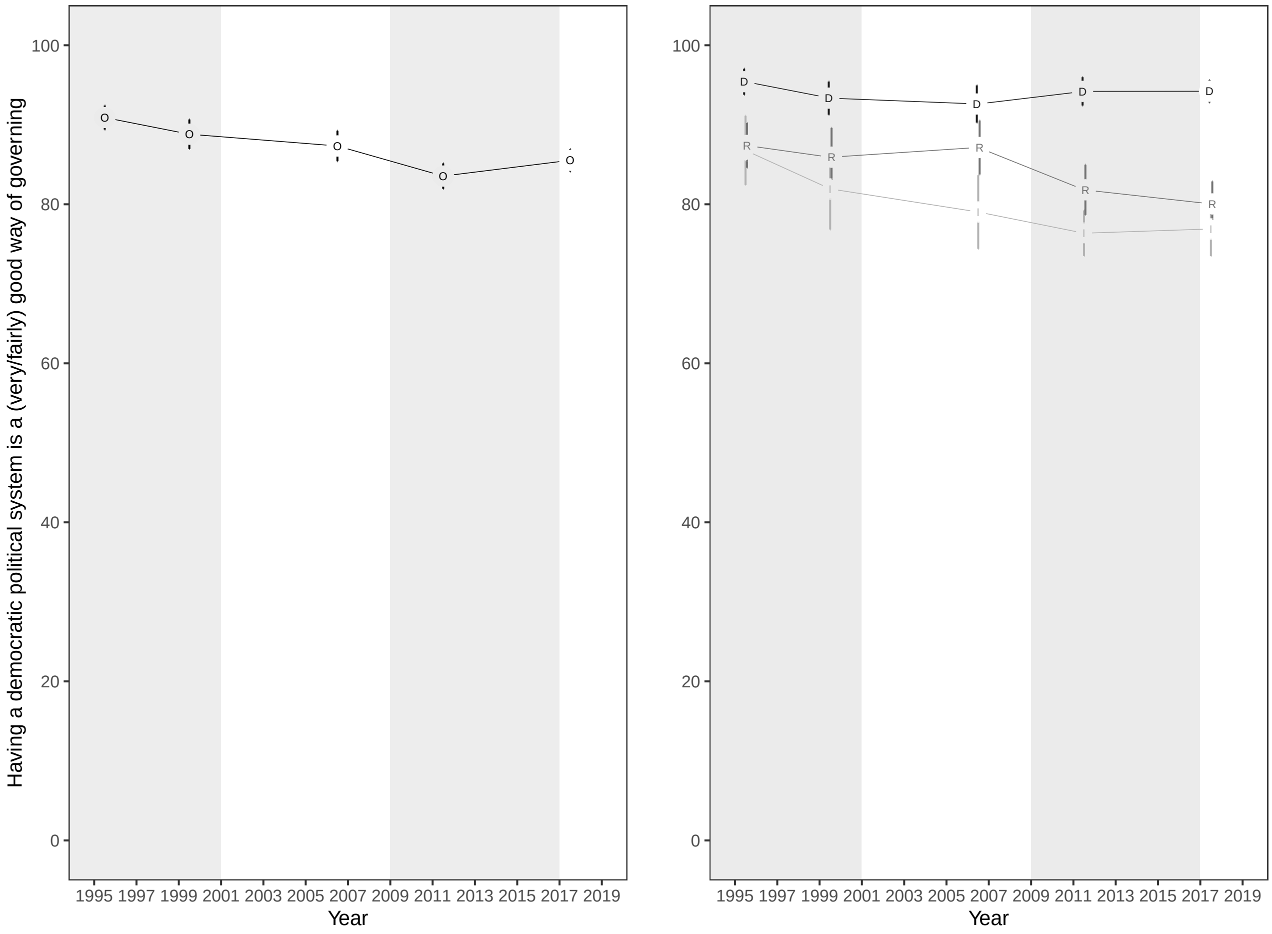

Figure SF1 (on the left) summarizes (o)verall numbers. **Figure SF2** (on the right) summarizes (R)epublicans, (D)emocrats, and (I)ndependents (including everyone who did not name Republicans or Democrats) measured as their party of choice in a hypothetical national election ”tomorrow”. Error bars denote +/- 2 standard deviations approximating 95% confidence intervals. Democratic administrations with gray background, Republican ones with white. Note that while Republicans dipped after the election of Obama (in 2007), their numbers never went below independents whose decrease was mild and more steady. Further World Values Survey documentation can be found at https://www.worldvaluessurvey.org/

Supplementary Figures and Table: Additional Americas Barometer Results (with fewer waves)

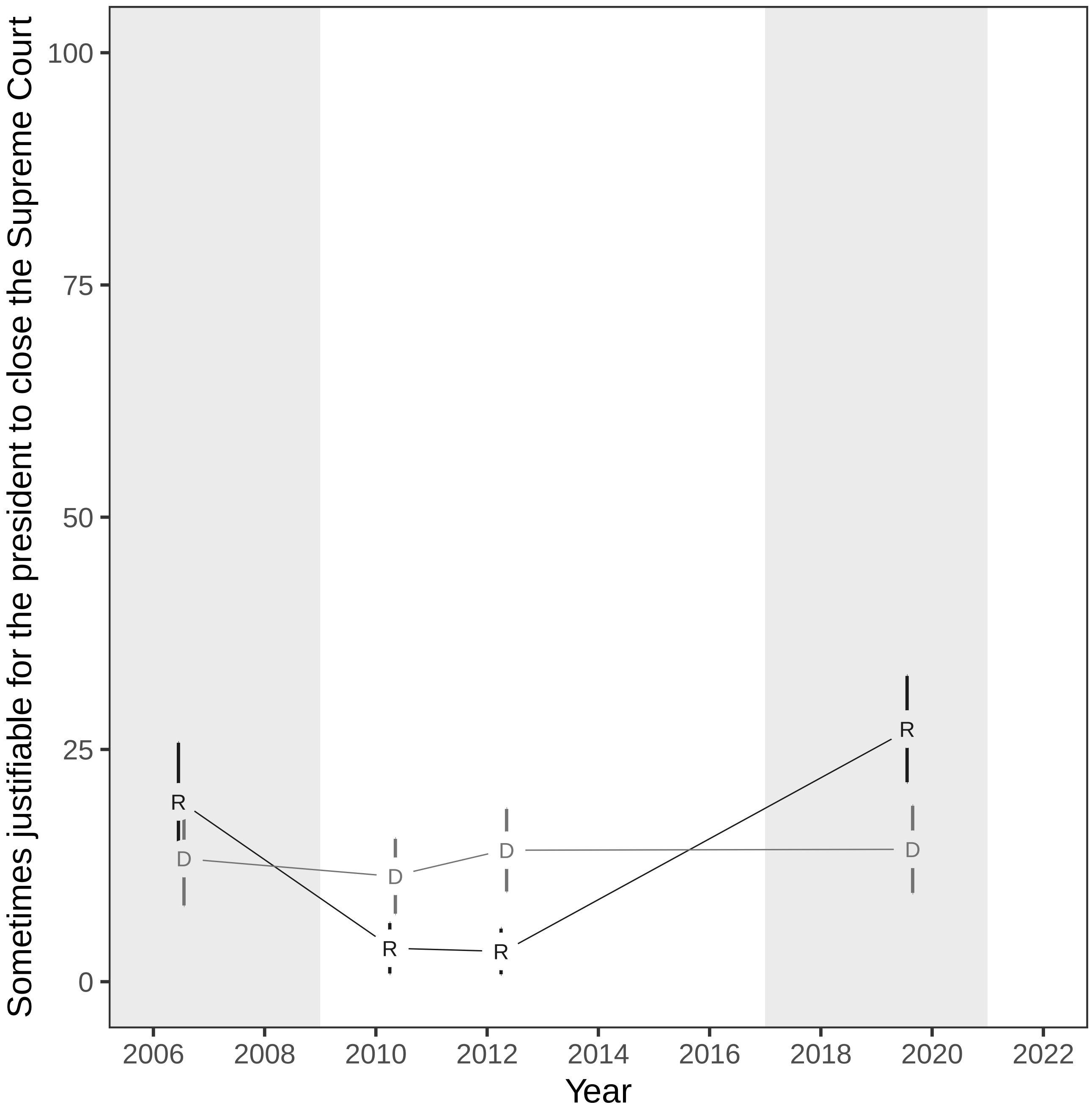

Figure SF3: % of agreement with the statement for (R)epublicans in black, (D)emocrats in gray. Republican administrations with gray background, Democratic ones with white. Error bars denote +/- 2 standard deviations approximating 95% confidence intervals.

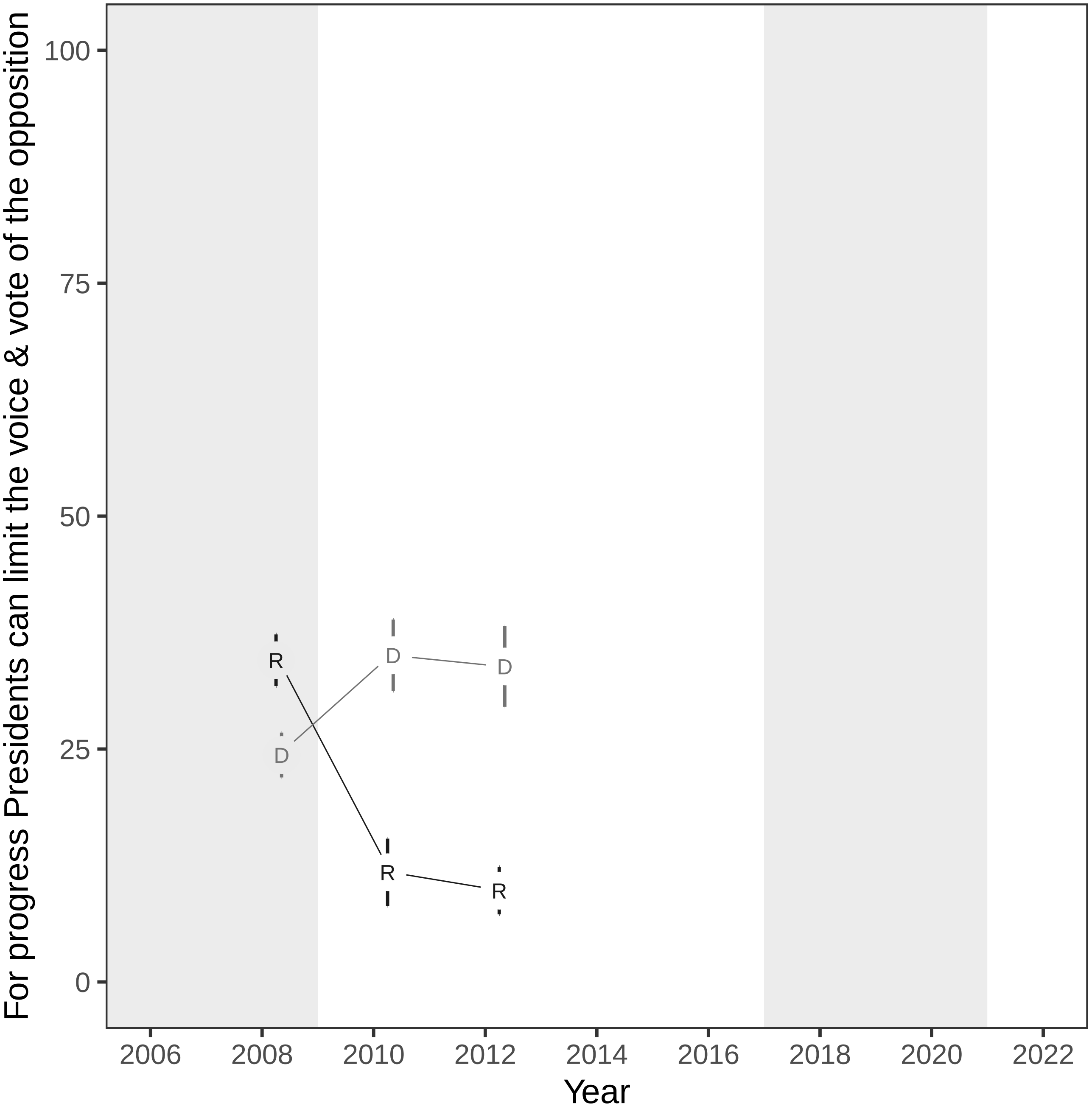

Figure SF4: Average levels (recoded from 7 point scale to 0 – 100) for (R)epublicans in black, (D)emocrats in gray. Republican administrations in gray background, Democratic ones with white. Error bars denote +/- 2 standard deviations approximating 95% confidence intervals.

Table ST1: Numerical Information for Figure 1, ST3, and ST4

Year	Close Congress	Close Congress SE	Close Supreme Court	Close Supreme Court SE	Limit Opposition	Limit Opposition SE	Party
2006.5	29.03	3.66	19.35	3.18			R
2008.3					34.53	1.39	R
2010.3	3.50	1.34	3.59	1.36	11.78	1.80	R
2012.3	3.75	1.32	3.26	1.23	9.80	1.27	R
2014.6	6.52	2.00					R
2017.5	27.19	2.39					R
2019.6	48.40	3.33	27.19	2.86			R
2021.6	14.38	1.93					R
2006.5	10.61	2.31	13.26	2.53			D
2008.3					24.36	1.20	D
2010.3	12.98	2.13	11.35	2.01	35.07	1.91	D
2012.3	22.71	2.67	14.15	2.22	33.86	2.16	D
2014.6	37.18	2.89					D
2017.5	13.10	1.49					D
2019.6	13.25	2.20	14.25	2.35			D
2021.6	20.36	1.63					D

Supplementary Tables and Figures: Original Data Collection Figure (2) with Independents and Numerical Information

Table ST2: Numerical Results for Figure 2

Group	Question	Proportion	n	SE
Democrats Under Biden (D)	Legislature to Change Size of Supreme Court (mr)	35.52	580	1.99
Democrats Under Biden (D)	Majority Party Appoints Lifetime Judges (mr)	42.27	582	2.05
Democrats Under Biden (D)	Majority Party to Draw Districts (mr)	42.78	582	2.05
Democrats Under Biden (D)	Government to Purge Voter Rolls (mr)	39.00	582	2.02
Democrats Under Biden (D)	President to Govern by Executive Order (er)	46.90	580	2.07
Democrats Under Biden (D)	President Should Do What Ppl Want (vs Follow the Law) (er)	35.46	581	1.98
Democrats Under Biden (D)	President Should Not Be Constrained by Congress and Courts (er)	37.63	582	2.01
Democrats Under Biden (D)	No Presidential Term Limits (er)	17.35	582	1.57
Democrats Under Biden (D)	Governors Allowed to Ban Protest (cl)	31.95	579	1.94
Democrats Under Biden (D)	Governors to Prosecute Journalists (cl)	37.89	578	2.02
Democrats Under Biden (D)	Governors Ban Religious Symbols (cl)	29.46	577	1.90
Democrats Under Biden (D)	Elected Officials Disobey Biased Courts (rl)	24.31	580	1.78
Democrats Under Biden (D)	Use Foreign Help in Campaign (rl)	31.66	578	1.93
Democrats Under Biden (D)	Candidates Need Not Respect Election Results (rl)	22.41	580	1.73
Democrats Under Biden (D)	President to Disqualify Candidates in Elections (cl)	46.18	576	2.08
Democrats Under Biden (D)	President Should Be Immune from Prosecution (rl)	18.97	580	1.63
Republicans Under Biden (D)	Legislature to Change Size of Supreme Court (mr)	19.75	476	1.82
Republicans Under Biden (D)	Majority Party Appoints Lifetime Judges (mr)	38.66	476	2.23
Republicans Under Biden (D)	Majority Party to Draw Districts (mr)	30.67	476	2.11
Republicans Under Biden (D)	Government to Purge Voter Rolls (mr)	48.32	476	2.29
Republicans Under Biden (D)	President to Govern by Executive Order (er)	25.63	476	2.00
Republicans Under Biden (D)	President Should Do What Ppl Want (vs Follow the Law) (er)	25.58	473	2.01
Republicans Under Biden (D)	President Should Not Be Constrained by Congress and Courts (er)	22.48	476	1.91
Republicans Under Biden (D)	No Presidential Term Limits (er)	18.07	476	1.76
Republicans Under Biden (D)	Governors Allowed to Ban Protest (cl)	36.63	475	2.21
Republicans Under Biden (D)	Governors to Prosecute Journalists (cl)	30.17	474	2.11
Republicans Under Biden (D)	Governors Ban Religious Symbols (cl)	18.74	475	1.79
Republicans Under Biden (D)	Elected Officials Disobey Biased Courts (rl)	22.69	476	1.92
Republicans Under Biden (D)	Use Foreign Help in Campaign (rl)	25.11	474	1.99
Republicans Under Biden (D)	Candidates Need Not Respect Election Results (rl)	45.88	473	2.29
Republicans Under Biden (D)	President to Disqualify Candidates in Elections (cl)	36.92	474	2.22
Republicans Under Biden (D)	President Should Be Immune from Prosecution (rl)	17.12	473	1.73
Democrats Under Trump (R)	Legislature to Change Size of Supreme Court (mr)	23.76	804	1.50
Democrats Under Trump (R)	Majority Party Appoints Lifetime Judges (mr)	32.92	808	1.65
Democrats Under Trump (R)	Majority Party to Draw Districts (mr)	35.15	808	1.68
Democrats Under Trump (R)	Government to Purge Voter Rolls (mr)	33.91	808	1.67
Democrats Under Trump (R)	President to Govern by Executive Order (er)	30.86	794	1.64
Democrats Under Trump (R)	President Should Do What Ppl Want (vs Follow the Law) (er)	27.97	801	1.59
Democrats Under Trump (R)	President Should Not Be Constrained by Congress and Courts (er)	21.61	805	1.45
Democrats Under Trump (R)	No Presidential Term Limits (er)	20.50	805	1.42
Democrats Under Trump (R)	Governors Allowed to Ban Protest (cl)	28.09	801	1.59
Democrats Under Trump (R)	Governors to Prosecute Journalists (cl)	30.16	799	1.62
Democrats Under Trump (R)	Governors Ban Religious Symbols (cl)	23.28	799	1.50
Democrats Under Trump (R)	Elected Officials Disobey Biased Courts (rl)	24.00	800	1.51
Democrats Under Trump (R)	Use Foreign Help in Campaign (rl)	27.41	799	1.58
Democrats Under Trump (R)	Candidates Need Not Respect Election Results (rl)	29.61	797	1.62
Democrats Under Trump (R)	President to Disqualify Candidates in Elections (cl)	24.56	798	1.52
Democrats Under Trump (R)	President Should Be Immune from Prosecution (rl)	12.55	797	1.17
Republicans Under Trump (R)	Legislature to Change Size of Supreme Court (mr)	15.66	747	1.33
Republicans Under Trump (R)	Majority Party Appoints Lifetime Judges (mr)	44.21	751	1.81
Republicans Under Trump (R)	Majority Party to Draw Districts (mr)	38.22	751	1.77
Republicans Under Trump (R)	Government to Purge Voter Rolls (mr)	47.00	751	1.82
Republicans Under Trump (R)	President to Govern by Executive Order (er)	65.30	732	1.76
Republicans Under Trump (R)	President Should Do What Ppl Want (vs Follow the Law) (er)	34.45	743	1.74
Republicans Under Trump (R)	President Should Not Be Constrained by Congress and Courts (er)	49.53	747	1.83
Republicans Under Trump (R)	No Presidential Term Limits (er)	15.53	747	1.33
Republicans Under Trump (R)	Governors Allowed to Ban Protest (cl)	50.74	739	1.84
Republicans Under Trump (R)	Governors to Prosecute Journalists (cl)	39.57	738	1.80
Republicans Under Trump (R)	Governors Ban Religious Symbols (cl)	26.59	741	1.62
Republicans Under Trump (R)	Elected Officials Disobey Biased Courts (rl)	22.51	742	1.53
Republicans Under Trump (R)	Use Foreign Help in Campaign (rl)	34.68	741	1.75
Republicans Under Trump (R)	Candidates Need Not Respect Election Results (rl)	30.73	742	1.69
Republicans Under Trump (R)	President to Disqualify Candidates in Elections (cl)	48.24	740	1.84
Republicans Under Trump (R)	President Should Be Immune from Prosecution (rl)	33.24	743	1.73

Table ST3: Numerical Results for Figure SF5 (Figure 2 with independents excluded)

Group	Question	Proportion	n	SE
Democrats Under Biden (D)	Legislature to Change Size of Supreme Court (mr)	33.89	416	2.32
Democrats Under Biden (D)	Majority Party Appoints Lifetime Judges (mr)	46.28	417	2.44
Democrats Under Biden (D)	Majority Party to Draw Districts (mr)	46.76	417	2.44
Democrats Under Biden (D)	Government to Purge Voter Rolls (mr)	40.29	417	2.40
Democrats Under Biden (D)	President to Govern by Executive Order (er)	52.29	415	2.45
Democrats Under Biden (D)	President Should Do What Ppl Want (vs Follow the Law) (er)	37.50	416	2.37
Democrats Under Biden (D)	President Should Not Be Constrained by Congress and Courts (er)	41.97	417	2.42
Democrats Under Biden (D)	No Presidential Term Limits (er)	17.27	417	1.85
Democrats Under Biden (D)	Governors Allowed to Ban Protest (cl)	32.61	414	2.30
Democrats Under Biden (D)	Governors to Prosecute Journalists (cl)	41.55	414	2.42
Democrats Under Biden (D)	Governors Ban Religious Symbols (cl)	33.74	412	2.33
Democrats Under Biden (D)	Elected Officials Disobey Biased Courts (rl)	24.52	416	2.11
Democrats Under Biden (D)	Use Foreign Help in Campaign (rl)	34.87	413	2.34
Democrats Under Biden (D)	Candidates Need Not Respect Election Results (rl)	22.12	416	2.03
Democrats Under Biden (D)	President to Disqualify Candidates in Elections (cl)	48.18	411	2.46
Democrats Under Biden (D)	President Should Be Immune from Prosecution (rl)	20.72	415	1.99
Republicans Under Biden (D)	Legislature to Change Size of Supreme Court (mr)	16.97	277	2.26
Republicans Under Biden (D)	Majority Party Appoints Lifetime Judges (mr)	38.99	277	2.93
Republicans Under Biden (D)	Majority Party to Draw Districts (mr)	31.41	277	2.79
Republicans Under Biden (D)	Government to Purge Voter Rolls (mr)	53.43	277	3.00
Republicans Under Biden (D)	President to Govern by Executive Order (er)	27.44	277	2.68
Republicans Under Biden (D)	President Should Do What Ppl Want (vs Follow the Law) (er)	24.45	274	2.60
Republicans Under Biden (D)	President Should Not Be Constrained by Congress and Courts (er)	23.10	277	2.53
Republicans Under Biden (D)	No Presidential Term Limits (er)	16.97	277	2.26
Republicans Under Biden (D)	Governors Allowed to Ban Protest (cl)	39.13	276	2.94
Republicans Under Biden (D)	Governors to Prosecute Journalists (cl)	30.80	276	2.78
Republicans Under Biden (D)	Governors Ban Religious Symbols (cl)	21.38	276	2.47
Republicans Under Biden (D)	Elected Officials Disobey Biased Courts (rl)	20.22	277	2.41
Republicans Under Biden (D)	Use Foreign Help in Campaign (rl)	25.00	276	2.61
Republicans Under Biden (D)	Candidates Need Not Respect Election Results (rl)	48.00	275	3.01
Republicans Under Biden (D)	President to Disqualify Candidates in Elections (cl)	35.64	275	2.89
Republicans Under Biden (D)	President Should Be Immune from Prosecution (rl)	14.91	275	2.15
Democrats Under Trump (R)	Legislature to Change Size of Supreme Court (mr)	22.74	563	1.77
Democrats Under Trump (R)	Majority Party Appoints Lifetime Judges (mr)	32.09	564	1.97
Democrats Under Trump (R)	Majority Party to Draw Districts (mr)	36.52	564	2.03
Democrats Under Trump (R)	Government to Purge Voter Rolls (mr)	34.93	564	2.01
Democrats Under Trump (R)	President to Govern by Executive Order (er)	30.94	556	1.96
Democrats Under Trump (R)	President Should Do What Ppl Want (vs Follow the Law) (er)	28.16	561	1.90
Democrats Under Trump (R)	President Should Not Be Constrained by Congress and Courts (er)	21.89	562	1.74
Democrats Under Trump (R)	No Presidential Term Limits (er)	19.57	562	1.67
Democrats Under Trump (R)	Governors Allowed to Ban Protest (cl)	27.73	559	1.89
Democrats Under Trump (R)	Governors to Prosecute Journalists (cl)	30.47	558	1.95
Democrats Under Trump (R)	Governors Ban Religious Symbols (cl)	24.60	557	1.82
Democrats Under Trump (R)	Elected Officials Disobey Biased Courts (rl)	21.01	557	1.73
Democrats Under Trump (R)	Use Foreign Help in Campaign (rl)	26.16	558	1.86
Democrats Under Trump (R)	Candidates Need Not Respect Election Results (rl)	27.70	556	1.90
Democrats Under Trump (R)	President to Disqualify Candidates in Elections (cl)	24.37	558	1.82
Democrats Under Trump (R)	President Should Be Immune from Prosecution (rl)	12.21	557	1.39
Republicans Under Trump (R)	Legislature to Change Size of Supreme Court (mr)	13.98	522	1.52
Republicans Under Trump (R)	Majority Party Appoints Lifetime Judges (mr)	47.43	525	2.18
Republicans Under Trump (R)	Majority Party to Draw Districts (mr)	41.90	525	2.15
Republicans Under Trump (R)	Government to Purge Voter Rolls (mr)	47.43	525	2.18
Republicans Under Trump (R)	President to Govern by Executive Order (er)	71.54	513	1.99
Republicans Under Trump (R)	President Should Do What Ppl Want (vs Follow the Law) (er)	37.19	519	2.12
Republicans Under Trump (R)	President Should Not Be Constrained by Congress and Courts (er)	52.49	522	2.19
Republicans Under Trump (R)	No Presidential Term Limits (er)	14.18	522	1.53
Republicans Under Trump (R)	Governors Allowed to Ban Protest (cl)	51.35	518	2.20
Republicans Under Trump (R)	Governors to Prosecute Journalists (cl)	43.11	515	2.18
Republicans Under Trump (R)	Governors Ban Religious Symbols (cl)	29.92	518	2.01
Republicans Under Trump (R)	Elected Officials Disobey Biased Courts (rl)	21.77	519	1.81
Republicans Under Trump (R)	Use Foreign Help in Campaign (rl)	36.49	518	2.12
Republicans Under Trump (R)	Candidates Need Not Respect Election Results (rl)	29.29	519	2.00
Republicans Under Trump (R)	President to Disqualify Candidates in Elections (cl)	52.13	516	2.20
Republicans Under Trump (R)	President Should Be Immune from Prosecution (rl)	34.30	519	2.08

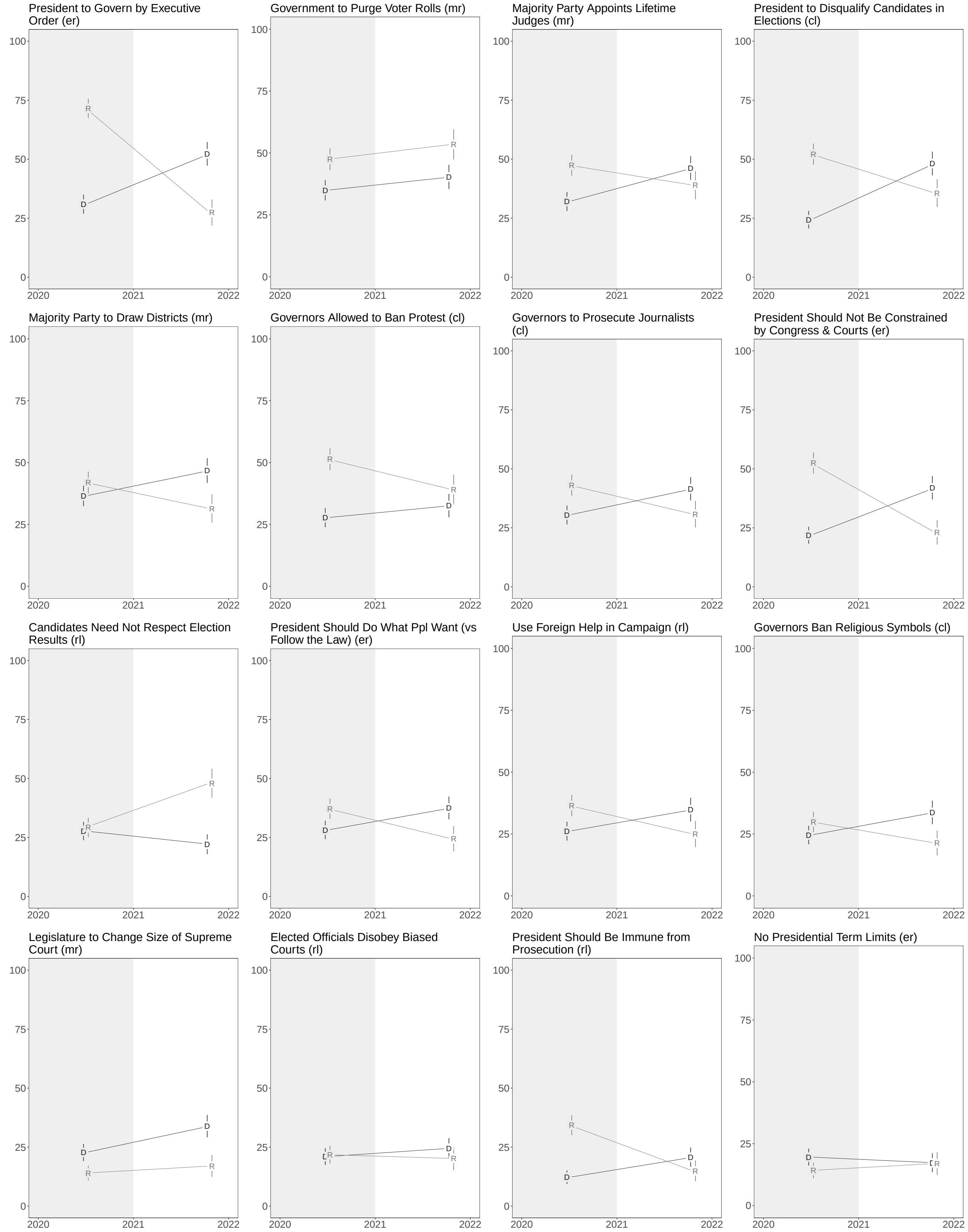

Figure SF5: Figure 2 with Independents Excluded % of agreement with the liberal democracy eroding statement for (R)epublicans in black, (D)emocrats in gray. Republican (Trump) administration with gray background, Democratic (Biden) administration with white. Error bars denote +/- 2 standard deviations approximating 95% confidence intervals.
